# A Mathematical Model for Interpretable Clinical Decision Support with Applications in Gynecology

**DOI:** 10.1371/journal.pone.0034312

**Published:** 2012-03-29

**Authors:** Vanya M. C. A. Van Belle, Ben Van Calster, Dirk Timmerman, Tom Bourne, Cecilia Bottomley, Lil Valentin, Patrick Neven, Sabine Van Huffel, Johan A. K. Suykens, Stephen Boyd

**Affiliations:** 1 Department of Electrical Engineering (ESAT-SCD), Katholieke Universiteit Leuven, Leuven, Belgium; 2 IBBT - Future Health Department, Katholieke Universiteit Leuven, Leuven, Belgium; 3 Department of Development and Regeneration, University Hospitals Leuven, Leuven, Belgium; 4 Department of Obstetrics and Gynaecology, Imperial College London, Hammersmith Campus, London, United Kingdom; 5 Chelsea and Westminster Hospital, London, United Kingdom; 6 Department of Obstetrics and Gynecology, Skåne University Hospital Malmö, Lund University, Malmö, Sweden; 7 Multidisciplinary Breast Centre (MBC), University Hospitals Leuven, Leuven, Belgium; 8 Department of Gynaecological Oncology, University Hospitals Leuven, Leuven, Belgium; 9 Department of Electrical Engineering, Stanford University, Stanford, California, United States of America; Dana-Farber Cancer Institute, United States of America

## Abstract

**Background:**

Over time, methods for the development of clinical decision support (CDS) systems have evolved from interpretable and easy-to-use scoring systems to very complex and non-interpretable mathematical models. In order to accomplish effective decision support, CDS systems should provide information on how the model arrives at a certain decision. To address the issue of incompatibility between performance, interpretability and applicability of CDS systems, this paper proposes an innovative model structure, automatically leading to interpretable and easily applicable models. The resulting models can be used to guide clinicians when deciding upon the appropriate treatment, estimating patient-specific risks and to improve communication with patients.

**Methods and Findings:**

We propose the interval coded scoring (ICS) system, which imposes that the effect of each variable on the estimated risk is constant within consecutive intervals. The number and position of the intervals are automatically obtained by solving an optimization problem, which additionally performs variable selection. The resulting model can be visualised by means of appealing scoring tables and color bars. ICS models can be used within software packages, in smartphone applications, or on paper, which is particularly useful for bedside medicine and home-monitoring. The ICS approach is illustrated on two gynecological problems: diagnosis of malignancy of ovarian tumors using a dataset containing 3,511 patients, and prediction of first trimester viability of pregnancies using a dataset of 1,435 women. Comparison of the performance of the ICS approach with a range of prediction models proposed in the literature illustrates the ability of ICS to combine optimal performance with the interpretability of simple scoring systems.

**Conclusions:**

The ICS approach can improve patient-clinician communication and will provide additional insights in the importance and influence of available variables. Future challenges include extensions of the proposed methodology towards automated detection of interaction effects, multi-class decision support systems, prognosis and high-dimensional data.

## Introduction

Since the invention of the computer, people have acknowledged the possible advantages that computers might offer in clinical decision making. In 1960, Ledley and Lusted [1] summarized ideas on how computers could be used for medical data processing. Clinical Decision Support (CDS) systems have changed extensively since these days. Thanks to increased computing power and the collection and storage of medical data, mathematical models designed for decision making are based on increasingly complex formulations. As a result, the obtained models often perform better than in the early days. However, the price to be paid is the lower interpretability of the model by the user. The earliest CDS systems were problem-specific flowcharts designed by clinicians and encoded for use by a computer [2], whereas later systems are based on logistic regression models, artificial neural networks, support vector machines, among others. Although the possible use of computers in medical decision making was mentioned 50 years ago, CDS systems are still not widely applied nor accepted in clinical practice [3]–[6]. Potential features to make them more acceptable for clinical practice have been proposed by several authors [7]–[9]. Kawamoto [8] identified four features which are crucial for CDS systems: the system should (i) be provided to clinicians in an automatic way, without interfering with the workflow, (ii) provide decision support at the time and location of decision making, (iii) provide a recommendation and (iv) be implemented on a computer. However, there is no real consensus on the properties of CDS systems. While some suggest that they should be implemented on a computer, others state that prediction models should be taken to the bedside. Kattan [10] proposed the use of nomograms [11]–[19] as a paper-based CDS system. A nomogram is defined as a graphical tool representing a regression model, such that the user is able to calculate the patient-specific risk without the use of a calculator or computer, given certain variables (see Text S1). However, applications of nomograms in clinical practice are rare and mostly used within software applications. The problem of application and interpretation becomes even harder when using more advanced models. Musen [2] summarized this issue as: “There is no way that an observer can directly understand why an artificial neural network might reach a particular decision”. Although rule extraction methods have been proposed [20], the transformation of advanced mathematical models into a CDS system remains difficultly acceptable for clinicians. A summary of classification methods, their advantages and disadvantages together with their clinical use according to the current state-of-the art is given in Table 1. Logistic regression models are very popular in clinical decision making, mainly thanks to the simple model structure. However, they are mainly used within software implementations or within score systems that simplify the original model. Although this simplification is advantageous with respect to applicability, it is uncertain what the effects are on model performance due to this additional post-processing of the model. Nomograms are graphical tools that visualize logistic regression models and thus increase their interpretability. The use of nomograms is very easy when embedded in a software package. However, when used manually, this method is time-consuming and prone to errors of which the impact has, to our knowledge, never been studied. More advanced models such as artificial neural networks and (least-squares) support vector machines are very flexible models able to model non-linearities and interactions between covariates in an automatic way. Unfortunately, this reduces the model's interpretability. As a result, these types of models are only sporadically used in clinical decision making.

**Table 1:** Advantages and disadvantages of different classification methods in clinical decision making.

	LR^1^	Nomogramafter LR	Score systemafter LR	(LS-)SVM^2^non-additive kernelor ANN^3^	(LS-)SVMadditive kernel	Rule extraction after (LS-)SVM	ICS
Interpretability	+	++	+++	− −	+	−	+++
Speed when used manually	− −	−	+	− − −	−−	+	++
Communication to patients	−	−	++	− −	−	+	++
Usable by patients	−	+	++	− −	−	++	+++
Underlying model structure	simple	simple	simple	very flexible	flexible	flexible	flexible
Applicability							
*By hand*	−	+	++	− − −	− −	++	+++
*In software*	+	+	+	+	+	+	++
Post-processing^4^			yes			yes	no

1 Logistic regression.

2 (Least-squares) Support Vector Machine.

3 Artificial Neural Network.

4 Post-processing in order to obtain interpretable and easily applicable models.

It is the goal of this work to combine the advantages of non-linear modeling using advanced models with the easy applicability and interpretability of score systems [21] in such a way that the obtained models directly correspond to automatically generated questionnaires. The resulting models can be used in clinical practice in different ways: (i) paper- or computer-based, (ii) figure- or table-based, (iii) with or without additional colors for a more explicit visualization of the covariate effects. Depending on the user's preferences one modality can be chosen for implementation in clinical practice, which will increase the use of more flexible models and improve patient-doctor communication.

## Methods

### Mathematical modelling

Consider a problem where 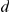
variables are measured for 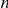
patients. We denote the variables of the 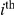 patient as 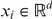. The 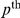 variable of this patient will be indicated as 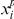. The outcome (benign or malignant) will be represented by 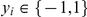. The mathematical model proposed by Vapnik [22] for linear classification (linear support vector machine) is formulated as follows
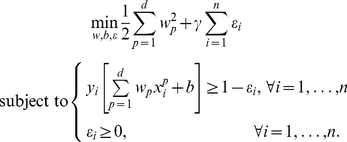
(1)In short, one tries to find the coefficients 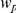 with 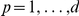 and a constant 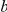, such that the model 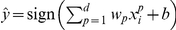 indicates what the estimated outcome for observation 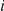 is. In practice, a tumor is predicted to be benign if 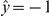 and malignant if 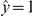. The other elements in equation (1) are included to ensure that the model performs well on other data than the data it is trained upon. The parameter 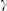 is a strictly positive constant, which enables to make a trade-off between tolerating misclassifications on the training data and having large coefficients 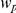. This method is extended towards non-linear classifiers by means of a feature map 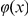, representing an unknown transformation function of the variables 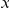.

The method that we present here involves three modifications with respect to the above model. The first two modifications involve the type of transformation 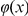
of the variables. A major problem for the application of models built using (1) is that the transformation can involve all variables. To enhance the interpretability, we will consider separate transformations for each covariate. In fact, the presented method is a special case of generalized additive models [23]. If for the problem at hand one is interested in specific interactions, or data in the literature suggest an interaction between two variables, one can include interaction terms as additional variables (see the example on prediction of non-viability in early pregnancies). The second modification in the transformation function implies that we impose the transformation to be a step function. Thus, the model is a generalized additive model with functional forms closely related to constant B-splines [24], [25]. The method itself will find out how many intervals are relevant, where these intervals should be located and how big the intervals should be. A third adaptation involves the sparsity of the model, implying that we want only a small number of variables to be included in the model, and in general, typically only a small number of intervals will be used in the step function for each variable. This feature is obtained within a convex optimization problem [26] by minimizing the total variation of the coefficient vector 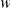 (see [27] and references therein). Inclusion of all above-mentioned adaptations leads to the following model:
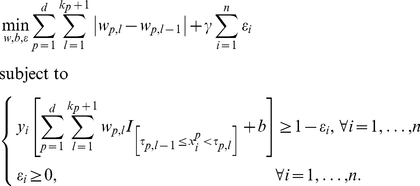
(2)In the above formulation, a number of 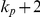 thresholds 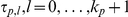 are included for variable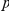, such that 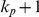 binary indicators 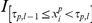, corresponding to 0/1 values represent one variable. Here, 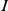 is the indicator function defined as: 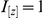 if 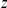 is true and zero otherwise. Note the link with compressed sensing [28] and constant B-splines [24], [25]. The weight 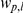 represents the effect on the risk of the variable 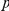 having a value within the 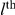 interval. For categorical variables, the binary indicators are indicating whether or not the value of the variable equals the first level, the second level, etc. For binary variables, the binary indicator indicates whether or not the value of the variable equals one. To achieve a very simple model including only a few intervals in the transformation of some of the variables, the absolute value of the difference between the coefficients is minimized (see (2)) [27]. After all the adaptations, model (2) retains the same loss function as model (1).

**Figure 1:**
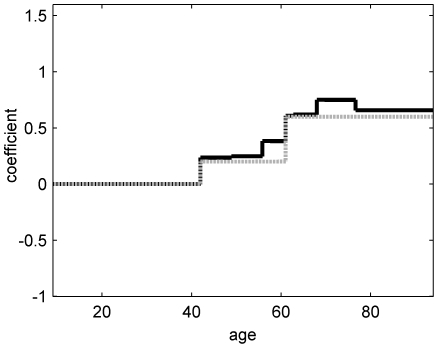
Illustration of the effect of iteratively reweighted 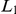
 **regularization.** The unweighted model results in the black solid functional form. After iteratively reweighted 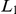 regularization, the estimated functional form becomes much sparser (see gray dashed line). Small and clinically irrelevant intervals are removed from the functional form.

A problem with the proposed method is that it can still lead to a solution including small intervals, which are often irrelevant for clinicians. In order to reduce the number of small intervals, equation (2) is iteratively reweighted as follows (see [29] for more information). First, equation (2) is solved. In a second step, weighting factors are calculated as
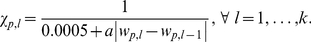
(3)By choosing the constant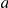, the amount of weighting, and thus the resulting number of selected variables and the number of intervals, can be controlled. Now, the weighted model
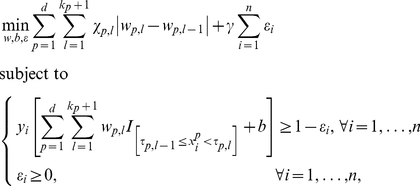
(4)is solved iteratively, until the differences between the estimated coefficients in subsequent iterations are bounded by a predefined small number (here 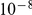). The result of the iteratively reweighted procedure is illustrated in Figure 1.

Although the result is easy to interpret, it is not yet easy to apply. We therefore propose to normalize the coefficients such that the absolute value of the smallest non-zero weight becomes 1. All other normalized coefficients are rounded to the nearest integer. Finally, the resulting score for any observation with variables 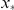 is given by
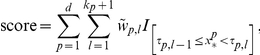
with 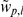 the normalized and rounded coefficients. The score is thus found by a weighted sum of binary values. Each binary operation corresponds to the question whether the covariate value lies within an interval. Since the resulting score is found as coding the variables by means of intervals, the method is referred to as the Interval Coded Score (ICS) index. Although the score will be higher for patients with higher risk, it is not an estimate of the risk. To obtain the risk, a link function, which links the final score with the risk, needs to be estimated. The link function is estimated by means of monotonic least squares support vector regression [30]–[32].

### Model selection

The area under the receiver operating curve (AUC) was used as a model selection criterion with 10-fold cross-validation. For estimation of the link function (risk prediction), the 10-fold cross-validated likelihood using a clinical kernel [33] was minimized. The optimal level of weighting for the iterative reweighting of the model was determined such that the model's performance (expressed by the AUC, the likelihood and the level of explained variance (R^2^_adj_) [34] in 5-fold cross-validation) was comparable with the performance of the unweighted model while selecting a minimal number of intervals.

### Statistical analysis

The performance of the model was expressed in terms of discrimination and calibration [34]. The discrimination performance was expressed by means of the AUC. The 95% confidence intervals were calculated using the bias corrected percentile bootstrap method based on 1,000 bootstrap samples [35]. Calibration performance was illustrated with calibration plots [36] and expressed by the ratio of the average predicted risk to the observed risk (the prevalence of the event). The average predicted risk in groups of at least 10% of the data were calculated, and plotted against the prevalence. Additionally, the R^2^_adj_ is reported. For the first illustration, clinicians were interested in the model's performance when using a cut-off on the estimated risk. Sensitivity, specificity, positive and negative likelihood ratios (LR^+^ and LR^−^) and the diagnostic odds ratio (DOR) [37] were reported based on the selected cut-off.

### Use and visualization

In order to visualize the resulting models for use in clinical practice, one needs to get an idea on who will use the model and in which circumstances. A first class of users will be clinicians, who may want to use it in an examination room, at the bedside of the patient, or during a consultation. The clinician might therefore opt for a computer-based application or an application on a portable device such as a smartphone. A second class of users might be the patients. Depending on the problem at hand, the model might be created from variables that the patient can calculate at home. Using a paper-based version, the patient will be able to check the evolution of his risk during treatment. A paper-based version might also be helpful for the clinician when explaining the results to a patient. Additionally, one might opt for a table or figure representation. Different possible implementations are discussed and illustrated below. Table 2 summarizes the properties of different implementations. Different computer-based alternatives are provided as Movie S1, Movie S2, Movie S3 and Text S1.

**Table 2:** Properties of different model implementations for clinical use.

Representation	Table or figure	Paper or software	Color representation	Illustrative example
1	Table	Paper	No	Table 3
2	Figure	Paper	No	Figure 1
3	Figure	Paper	No	Figure 2
4	Table	Software	No	Movie S1
5	Figure	Software	No	Movie S2
6	figure	Software	Yes	Movie S3

**Figure 2:**
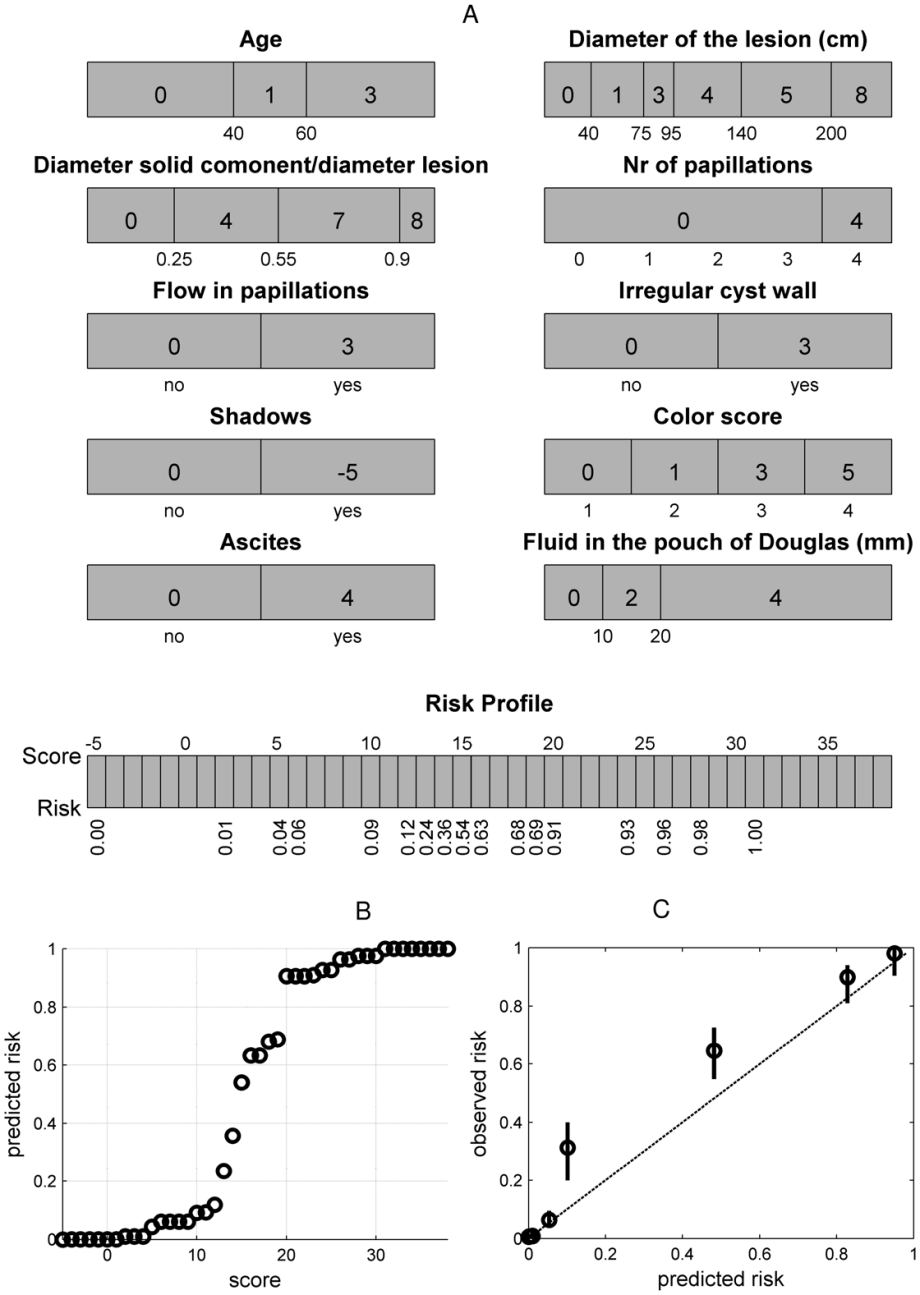
Application of the ICS approach to the diagnosis of the malignancy of adnexal masses. (a) Picture-based representation by means of bar charts (without color indications) representing the intervals in which the variable effect is estimated to be constant. The bottom bar represents the predicted risk associated with the final score, obtained by summing all contributions of all variables. A software implementation is provided as Movie S2 and Movie S3. (b) Estimated link function, linking the score with the risk of a malignant tumor. (c) Calibration of the ICS model on the test set. For each possible value of the predicted risk (some values were taken together in order to obtain at least 10% of the patients in each group), the observed percentage of malignancies is calculated (dots). A 95% confidence interval on the percentage of the observed malignancies is illustrated by means of the vertical lines.

**Figure 3:**
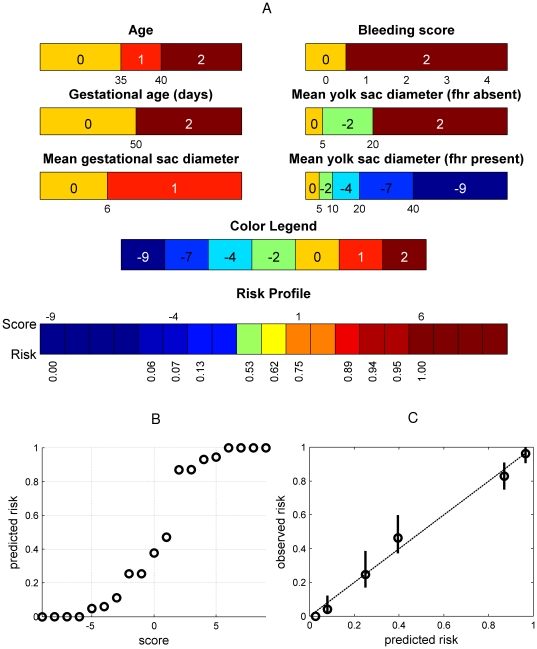
Application of the ICS approach to the prediction of non-viable pregnancies. (a) Picture-based representation by means of color bars, representing the intervals in which the variable effect is estimated to be constant. For each of the represented bars, the points corresponding to the value of the patient's covariates are obtained. The total score is obtained by summing all points. The color bar at the bottom represents the predicted risk associated with the final score. (b) Estimated link function, linking the score with the risk of a non-viable pregnancy at the end of the first trimester. (c) Calibration of the ICS model on the test set. For each possible value of the predicted risk (some values were taken together in order to obtain at least 10% of the patients in each group), the observed percentage of non-viable pregnancies is calculated (dots). A 95% confidence interval on the percentage of the observed non-viable pregnancies is illustrated by means of the vertical lines. Fhr: fetal heart rate.

Since the outcome of an ICS model consists of variable intervals and their corresponding weights, the model can be implemented in a user-friendly way. As illustrated in Table 3, each interval can be represented by means of simple yes/no questions. When the answer to the question is yes, the points to the right need to be added to the score. In Table 4, the final score is linked with the estimated risk. Alternatively, instead of questions, one could opt for a bar-representation, as illustrated in Figure 2. Each bar represents one variable. The bars are divided into different regions in which the corresponding points are denoted. Addition of the points corresponding to the patient's variable values yields the final score, which can again be linked with the estimated risk by means of the lowest part of the figure. Figure 3 shows an alternative, where colors are added such that regions with higher risks (i.e. higher points) are marked in red and regions with lower risks are indicated in blue. In a software implementation, tick boxes are provided, such that the user only has to indicate the correct interval for each covariate, whereafter the score and risk estimate are calculated (either automatically or manually).

**Table 3:** Illustration of a table-based representation which can be filled out by hand for the ICS index in diagnosing malignancies in adnexal masses.

Question	Points
*Is the patient*	
between 40 (included) and 60 years old?	1
older than 60 (included) years old?	3
*Is the maximal diameter of the lesion*	
between 40 (included) and 75 mm?	1
between 75 (included) and 95 mm?	3
between 95 (included) and 140 mm?	4
between 140 (included) and 200 mm?	5
larger than 200 (included) mm?	8
*Is the ratio of the solid component and the lesion*	
between 0.25 (included) and 0.55?	4
between 0.55 (included) and 0.9?	7
larger than 0.9 (included)?	8
*Are there*	
4 or more papillations?	4
*Is there any blood flow in the papillary structures?*	
yes	3
*Is the internal cyst wall*	
irregular?	3
*Are there acoustic shadows present?*	
yes	−5
*Is the color score equal to*	
2?	1
3?	3
4?	5
*Are there ascites present?*	
yes	4
*Is the measurement of Free fluid in the pouch of Douglas*	
between 10 (included) and 20 mm?	2
more than 20 mm (included)?	4

For each variable several questions, corresponding to the different variables intervals, are posed. If the answer to the question is yes, the points in the last column, need to be added to the score. The software based version is provided as Supporting Information: movie1.

**Table 4:** Link between the scores obtained from Table 3 and the estimate of the risk.

Score	Risk
≤1	<0.001
2 to 4	0.01
5	0.04
6 to 9	0.06
10 to 11	0.10
12	0.12
13	0.24
14	0.36
15	0.54
16 to 17	0.63
18 to 19	0.69
20 to 23	0.91
24 to 25	0.93
26 to 27	0.96
28 to 30	0.98
≥31	>0.99

## Results

The proposed method is illustrated on two diagnostic problems. For the first illustration, we used a dataset of 3,511 patients with an adnexal mass recruited within the framework of a large multi-center study using standardized examination techniques and definitions [38]. The model was trained on 2,514 patients from 9 centers and tested on 997 patients from 12 other centers. The second illustration involves prediction of the non-viability of pregnancies at 11–14 weeks, based upon variables known at the first ultrasound scan. This data included 1,435 patients from an Early Pregnancy Unit in London. More information on both datasets can be found in Text S1.

### Diagnosing malignancy of adnexal masses

The model was trained on 2,514 patients from 9 international centers and tested on 997 patients from 12 other centers. From the ICS model (Figure 2 and Tables 3 and 4), the clinician knows which questions need to be answered in order to estimate the risk that an adnexal mass is malignant. Consider a 56-year-old patient from the database, with a maximum lesion diameter of 133 mm, a ratio of the solid component diameter versus the lesion diameter of 0.74, more than three papillations with blood flow, an irregular cyst wall, no acoustic shadows, no ascites, a color score of 3 and 18 mm of free fluid in the pouch of Douglas. This patient receives one point for age, two points for the amount of free fluid, three each for the irregular cyst wall, the blood flow within the papillations, and the color score, four each for the lesion diameter and the number of papillations, and seven for the ratio of the solid component diameter and the lesion diameter. The total score is 27, which is translated into an estimated risk of malignancy of 0.96 by means of the estimated link function (see Figure 2(a–b) and Table 4). The true outcome for this patient was malignancy (clear cell carcinoma). The area under the receiver operating characteristic curve (AUC) on the training set was 0.95 (95% CI: 0.94–0.95). The AUC on the test set was 0.96 (95% CI: 0.94–0.97). The R^2^_adj_ equaled 0.73 on the training and 0.72 on the test set. The model underestimated the risk of malignancy on the test set (Figure 2(c)). However, this problem of underestimation was previously noted when using other models as well [39], and is probably caused by case-mix differences between the training and test sets. The ratio of the average predicted risk to the observed prevalence equaled 0.78 on the test set, confirming the underestimation of the risk. A decision cut-off was determined to obtain the best specificity for a sensitivity of at least 90%. This resulted in predicting a malignant tumor for a score of 10 or more. This cut-off resulted in a sensitivity and specificity of respectively 94% and 76% on the training set and of 93% and 86% on the test set. The positive and negative likelihood ratio on the test set were 6.53 and 0.078, respectively, corresponding to a DOR of 84.

### Predicting non-viability of pregnancies

For a second illustration a dataset of 1,435 women with a positive pregnancy test was available from an Early Pregnancy Unit in London. The data were randomly split into a training set of 955 patients and a test set of 480 patients. Little research [40], [41] has been done with respect to prediction of pregnancy non-viability. Moreover, this research concentrated on the prediction of ultimately non-viable pregnancies. We aimed at predicting non-viability at the end of the first trimester. Since the effect of gestational sac diameter was considered to be different for fetuses with or without a visualized heart rate, an interaction between these variables was considered during model building. The resulting ICS model (Figure 3) includes six variables: maternal age, bleeding score, gestational age, mean gestational sac diameter, mean yolk sac diameter and whether or not a fetal heart beat is seen. A higher score corresponds to a higher risk of a non-viable pregnancy. The AUC was 0.90 (95% CI = 0.88–0.92) on the training set and 0.92 (95% CI = 0.90–0.94) on the test set. The R^2^_adj_ equaled 0.64 and 0.62 on the training and test set, respectively. The ratio of the average predicted risk to the observed risk was 0.98 on the test set, indicating adequate calibration (Figure 3(c)).

### Comparison with other methods

The literature describes different models for diagnosing the malignancy of adnexal masses. A selection of these were used for comparison: the risk of malignancy index (RMI) [42], a logistic regression model (LR1) [39], a least-squares support vector machine (LS-SVM) and a relevance vector machine (RVM) [43], both with an RBF kernel. All models were tested on the same test set, but derived from different training sets. All compared models used feature selection techniques in order to reduce the number of necessary variables. Table 5 shows that the ICS model could compete with the other models. In addition, the ICS has the advantage of being highly interpretable and easily applicable. Since a nomogram is a graphical representation of a (logistic) regression model, the use of a computer-based nomogram would yield results identical to those of the LR1 model. However, a paper-based version of a nomogram would be prone to errors due to drawing lines that are not exactly vertical, and due to errors in reading the points. The ICS methodology solves these issues by indicating in which areas the risk estimates are robust to these errors (since risks are assumed to be constant over intervals) and by visualizing the awarded points for each interval as obtained from the estimated model.

**Table 5:** Summary of the performance of previously built models and the ICS model for the prediction of malignancy of adnexal masses.

Model	AUC (95% CI)	Sensitivity	Specificity	LR+	LR−	DOR (95% CI)
RMI	0.911 (0.880–0.935)	67.5	94.6	12.60	0.343	37 (24–57)
LR1	0.956 (0.939–0.968)	92.2	86.5	6.84	0.091	75 (46–125)
LS-SVM RBF	0.954 (0.935–0.967)	89.4	89.9	8.85	0.118	75 (47–120)
RVM RBF	0.951 (0.933–0.965)	90.6	87.7	7.39	0.107	69 (43–111)
ICS	0.958 (0.943–0.969)	93.3	85.7	6.53	0.078	84 (51–150)

The measures are calculated on the test set of 997 patients from external centers. Except for the AUC, all measures were calculated using the cut-off mentioned in the original paper.

In a second study, we compared the ICS approach with the classical score system approach, i.e. score systems obtained by approximating a logistic regression model (see [21]). In the latter case, a logistic regression model is built in a first step. Then, in order to make the model easy to use, the variables are divided into consecutive intervals that are chosen by the user. Within each interval, the effect for the midpoint of the interval is applied. Although both methods obtain the same type of score system, the results are quite different. The major difference lies in the fact that in the second model, the intervals are set by the user. Therefore, within the model building step, no control exists over the information that might be lost by choosing intervals. On the contrary, the ICS method is especially designed to select, already during the phase of model estimation, those intervals that retain as much information as possible. To illustrate this point, we built for both applications two classical score systems based on a logistic regression model that used the variables selected with the ICS methodology. The first score system used a high number of consecutive intervals for continuous variables (using intervals of 5 years for age, 10 mm for lesion and gestational sac diameter, 0.1 for the ratio, 15 mm for free fluid, 10 days for gestational age and 1.5 mm for yolk sac diameter), the second score system used a smaller number of consecutive intervals for continuous variables (using intervals of 10 years for age, 30 mm for lesion and 20 mm for gestational sac diameter, 0.25 for the ratio, 30 mm for free fluid, 20 days for gestational age and 3 mm for yolk sac diameter). The results indicate that ICS performed comparable to or better than both classical score systems while using fewer intervals (see Tables 6 and 7). Due to the fact that the user has to set the intervals, information might be lost when reducing the number of intervals in other scoring systems approaches. The ICS approach is able to obtain models with fewer intervals than other approaches by incorporating an interval selection procedure during model training, without affecting the model's performance.

**Table 6:** Summary of the test set performance of the ICS-based score system and two classical score systems (M1 and M2) for the prediction of malignancy of adnexal masses.

Model	AUC (95% CI)	R^2^_adj_	# intervals
ICS	0.958 (0.943–0.969)	0.72	30
LR	0.963 (0.947–0.974)	0.73	
M1	0.961 (0.944–0.971)	0.71	57
M2	0.933 (0.911–0.949)	0.58	36

The classical score systems are based on a logistic regression model using the variables selected with ICS (LR). In a second step, the variables are manually divided into intervals. M1 uses a high number of intervals for continuous variables, M2 uses fewer intervals. The ICS approach is able to obtain good performance using a small number of intervals. The classical score systems are able to obtain good performance provided that a large number of intervals is considered.

**Table 7:** Summary of the test set performance of the ICS-based score system and two classical score systems (M1 and M2) for the prediction of non-viability of pregnancies.

Model	AUC (95% CI)	R^2^_adj_	# intervals
ICS	0.924 (0.897–0.942)	0.62	17
LR	0.940 (0.916–0.957)	0.69	
M1	0.897 (0.872–0.922)	0.65	31
M2	0.788 (0.749–0.823)	0.36	23

The classical score systems are based on a logistic regression model using the variables selected with ICS (LR). In a second step, the variables are manually divided into intervals. M1 uses a high number of intervals for continuous variables, M2 uses fewer intervals. The ICS approach is able to obtain good performance using a small number of intervals. The classical score systems are able to obtain good performance provided that a large number of intervals is considered.

## Discussion

This paper proposes a new methodology in order to bridge the gap between advanced mathematical diagnostic models and their applicability in clinical practice. This gap is the consequence of increasingly complex models which can no longer be interpreted nor applied by clinicians. Where others proposed to approximate advanced models by means of score systems or rules in a post-processing step, our approach starts from a model representation which is interpretable and usable in a clinical setting. By building an additive model with constant effects within intervals, the result can be represented as a questionnaire or score chart. However, as opposed to other score system approaches, the ICS enables better control of possible information loss since the number, position, and length of the intervals are automatically optimized by the model.

The ICS model presented here can be used in daily clinical practice to solve a broad range of clinical questions. The class of binary classification problems can be solved with the current model equations of the ICS methodology. The areas of application include, but are not restricted to, diagnosing tumor malignancy, detecting abnormalities in biomedical signals, estimating the risk of cardiovascular disease, diabetes, pregnancy failure, tumor recurrence within 5 years after surgery, estimating the therapeutic effect of different therapies, and detecting predictive factors for various conditions. Thanks to the different implementation possibilities, the model and the resulting risk estimate or diagnosis can be presented to the patient in a more understandable way than any existing method. The improved interpretability in combination with good model performance enables a better doctor-patient communication which is becoming increasingly important in the age of informed patient decision making. In particular, the color-based representation can be suitable for this task. Patients will easily understand that the addition of a variable value within a red area increases while a value within a blue area decreases their risk.

In order for the ICS methodology to become a widely applicable model, extensions to model architectures for problem settings other than binary classification are needed. Firstly, the additive model structure can be expanded such that problems with more complex covariate effects can be modeled as well. The current equations can be extended to an ANOVA structure, where interactions between different covariates can be further included. Where the current model is able to include interactions that are known from the literature, an ANOVA extension will make it possible to include additional interactions.

A second extension involves clinical classification problems with more than two outcomes (multi-class problems). Although clinical multi-class problems are most often reduced to binary classification problems, only multi-class models can give the most appropriate answer to the problem. Adnexal tumors are often categorized as being benign or malignant, whereas four or more categories could be considered, such as benign, borderline, primary invasive and metastatic [44]. Since the optimal management depends on the type of malignancy, a model taking all these categories together is sub-optimal. In contrast with binary logistic regression models, multi-class logistic regression models are only sporadically used in medical problem settings, the reason being the reduced model interpretability. By extending the current ICS methodology to multi-class classifiers, a tool can be provided that enables the development of a more appropriate multi-class model structure which remains interpretable and easily applicable.

A third extension of the ICS methodology relates to disease prognosis. Survival studies are very common in clinical research. A few examples are the prognosis of primary operable breast cancer patients after surgery, the prognosis after organ transplantations, the prognosis of severely burnt patients and the prognosis of patients after a stroke. By incorporating the ICS methodology in flexible models for the analysis of survival data [45], easily applicable score systems for survival analysis can be developed.

A last challenge concerns the increasing number of high-dimensional data, such as genomics and proteomics data. The current ICS model equations are able to deal with clinical datasets, containing a large number of patients and a moderate number of covariates. In order to apply this model to high-dimensional data, specialized optimization algorithms will need to be developed. Further research on the ICS methodology together with preprocessing and feature selection algorithms for high-dimensional data, are needed for this purpose.

## Supporting Information

Supporting Information S1 Detailed description of the data, Table S1 (Description of the variables of the adnexal mass data set considered in this work), Table S2 (Description of the variables of the pregnancy data set considered in this work.), Illustration of the use of a nomogram for ovarian cancer diagnosis, Fig. S1 (Nomogram for the diagnosis of ovarian cancer derived from a logistic regression model), Illustration of a model implementation created with the ICS methodology, Software.
(PDF)

Movie S1 Software implementation of the table-based representation of the ICS model for diagnosing adnexal masses.
(MOV)

Movie S2 Software implementation of the figure-based representation of the ICS model for diagnosing adnexal masses, without color indication.
(MOV)

Movie S3 Software implementation of the figure-based representation of the ICS model for diagnosing adnexal masses, with color indications.
(MOV)
