# Supplementary material for: A Mathematical Model for Interpretable Clinical Decision Support with Applications in Gynecology
Source: PLoS One. 2012 Mar 29;7(3):e34312. doi: 10.1371/journal.pone.0034312 (PMC3315538; doi:10.1371/journal.pone.0034312)
Supplement: Supporting Information S1 — Detailed description of the data, Table S1 (Description of the variables of the adnexal mass data set considered in this work), Table S2 (Description of the variables of the pregnancy data set considered in this work.), Illustration of the use of a nomogram for ovarian cancer diagnosis, Fig. S1 (Nomogram for the diagnosis of ovarian cancer derived from a logistic regression model), Illustration of a model implementation created with the ICS methodology, Software. (PDF) [file pone.0034312.s001.pdf]

A Mathematical Model for Interpretable Clinical Decision Support with Applications in Gynecology

Vanya M.C.A. Van Belle[1,2], Ben Van Calster[1,2,3], Dirk Timmerman[3], Tom Bourne[3,4], Cecilia Bottomley[5], Lil Valentin[6], Patrick Neven[7,8], Sabine Van Huffel[1,2], Johan A.K. Suykens[1,2], and Stephen Boyd[9]

[1] Department of Electrical Engineering (ESAT-SCD), Katholieke Universiteit Leuven,Kasteelpark Arenberg 10, B-3001 Leuven, Belgium
[2] IBBT - Future Health Department, Katholieke Universiteit Leuven,Kasteelpark Arenberg 10, B-3001 Leuven, Belgium
[3] Department of Development and Regeneration, University Hospitals Leuven, Herestraat 49, B-3000 Leuven, Belgium
[4] Department of Obstetrics and Gynaecology, Imperial College London, Hammersmith Campus, Du Cane Road, London, W12 0HS, United Kingdom
[5] Chelsea and Westminster Hospital, 369 Fulham Road, London, SW10 9NH, United Kingdom
[6] Department of Obstetrics and Gynecology, Skåne University Hospital Malmö, Lund University, SE20502 Malmö, Sweden
[7] Multidisciplinary Breast Centre (MBC), University Hospitals Leuven,Herestraat 49, B-3000 Leuven, Belgium
[8] Department of Gynaecological Oncology, University Hospitals Leuven, Herestraat 49, B-3000 Leuven, Belgium
[9] Department of Electrical Engineering Stanford, Stanford University, CA 94305-9510 USA

Detailed description of the data

Diagnosis of the malignancy of adnexal masses

The dataset contains information on 3,511 patients with at least one overt persistent adnexal mass who received surgery. The data were recruited in three phases. In phase 1 1,066 patients in 9 centers were recruited between 1999 and 2002. In phase 1b three of these centers included 507 extra patients from the period 2002 to 2005. In phase 2 1,938 patients were recruited from 2005 to 2007. Seven of the centers from phase 1 and 12 new centers contributed to the second phase. Overall, the patients were recruited in 21 centers in 9 countries. Patients were included if the principal investigator from one of the participating centers assessed the mass. In case of multiple masses, the information from the most complex mass was included. Patients who were pregnant, refused transvaginal ultrasonography or did not undergo surgical removal of the mass within 120 days of the ultrasound examination were excluded. The variables considered in the analysis are summarized in Table S1. The outcome is the diagnosis of a tumor as benign or malignant (including borderline, primary invasive and metastatic tumors). Out of the 3,511 included tumors, 951 (27%) were malignant and 2,560 (73%) were benign.

Table S1: Description of the variables of the adnexal mass data set considered in this work.

variable	description	unit/level	type
age	age of patient	years	continuous
famhistovca	family members with history of ovarian cancer	binary (0 no; 1 yes)	binary
famhistbrca	nr of family members with history of breast cancer	count	ordinal
pershistovca	personal history of ovarian cancer	binary (0 no; 1 yes)	binary
pershistbrca	personal history of breast cancer	binary (0 no; 1 yes)	binary
parity	nr of deliveries	count	ordinal
hysterectomy	hysterectomy	binary (0 no; 1 yes)	binary
hormtherapy	current use of hormonal therapy	binary (0 no; 1 yes)	binary
bilateral	masses on both sides	binary (0 no; 1 yes)	binary

Continued on next page

Table S1 – continued from previous page

variable	description	unit/level	type
lesdmax	maximum diameter of lesion	mm	continuous
pain	pelvic pain during examination	binary (0 no; 1 yes)	binary
nrloculescat	number of locules	0 zero	ordinal
		1 one	
		2 two	
		3 three	
		4 four	
		5 five to ten	
		6 more than ten	
soldmax	max diameter of largest solid component	mm	continuous
papnr	nr of papillary structures	0 zero	ordinal
		1 one	
		2 two	
		3 three	
		4 more than three	
papheight	height of largest papillary structure	mm	continuous
papflow	presence of blood flow within papillary structures	binary (0 no; 1 yes)	binary
papsmooth	largest papillary structure is irregular	binary (0 no; 1 yes)	binary
wallreg	irregular internal cyst wall	binary (0 no; 1 yes)	binary
incomplseptum	presence of an incomplete septum	binary (0 no; 1 yes)	binary
shadows	presence of acoustic shadows	binary (0 no; 1 yes)	binary
echogenicity	echogenicity of cyst fluid	1 anechoic	categorical
		2 homogeneous low-level	
		3 ground glass	
		4 hemorrhagic	
		5 mixed	
		6 no cyst fluid	
colscore	color score of intratumoral blood flow	1 no blood flow	ordinal
		2 minimal	
		3 moderate	
		4 very strong	
venous	venous blood flow only	binary (0 no; 1 yes)	binary
ascites	presence of ascites	binary (0 no; 1 yes)	binary
fluid	amount of fluid in pouch of Douglas	mm	continuous

Prediction of non-viability of pregnancies

This data set is a prospective observational cohort of all women attending a single early pregnancy unit from January to October 2006. Inclusion criteria were: a positive pregnancy test and a gestational age of less than 12 weeks. Patients presenting with postpartum complications or complications from termination of pregnancy or recently diagnosed early pregnancy loss were not considered. Exclusion criteria were: multiple pregnancies, patients who appeared not to be pregnant and patients who underwent termination of pregnancy. Patients presenting with more than one pregnancy over the study period were included only once (first presenting pregnancy). A total of 1,435 pregnancies remained for analysis. The data were randomly divided into a training and test set, containing 955 and 480 patients, respectively. The outcome was defined as viable when a live fetus had been seen at the routine 11-14 week scan, performed after the initial scan. The outcome was non-viable whenever the pregnancy had resulted in a miscarriage, an ectopic pregnancy or a failed pregnancy of unknown location. Out of the 1,435 included pregnancies, 550 (38%) were non-viable and 885 (62%) were

viable. The variables considered in the experiments are summarized in Table S2. Missing values were imputed using distance-aided selection of donors [1].

Table S2: Description of the variables of the pregnancy data set considered in this work.

variable	description	unit/level	type
age	age of patient	years	continuous
pbac	bleeding score	0: no bleeding<br>1: light bleeding<br>2: moderate bleeding<br>3: heavy bleeding<br>4: bleeding with cloths	ordinal
gravida	number of pregnancies (including the present pregnancy)		ordinal
nodel24	number of deliveries after 24 weeks		ordinal
notop	number of termination of pregnancies		ordinal
noemis	number of early miscarriages		ordinal
nopul	previous pregnancies of unknown location	0: no<br>1: yes	binary
nolmis	previous late miscarriages	0: no<br>1: yes	binary
noect	previous ectopic pregnancies	0: no<br>1: yes	binary
gestdates	gestational age: days since start of last menstrual period	days	continuous
vas	pain score	cm	continuous
pain	is the patient in pain? (unspecified)	0: no<br>1: yes	binary
meangsd	mean gestational sac diameter	mm	continuous
meanysd	mean yolk sac diameter	mm	continuous
fhrseen	is a fetal heart rate seen?	0: no<br>1: yes	binary
fetus	is a fetus seen?	0: no<br>1: yes	binary
crl	crown-rump length	mm	continuous
regdates	did the patient report a regular cycle of 26 to 30 days?	0: no<br>1: yes	binary

Illustration of the use of a nomogram for ovarian cancer diagnosis

Figure S1 is a nomogram, illustrating a logistic regression model for diagnosing adnexal masses as benign or malignant. This model was proposed in [2]. The nomogram consists of several rulers. The first one is a reference ruler, the last two are rulers to obtain the results. The rulers in between correspond to each relevant variable. Consider a 70- year-old woman with an adnexal mass, without ascites, with flow in the papillary structures of the mass, a maximal diameter of the solid component of 25 mm, with an irregular cyst wall and no acoustic shadows. Looking at the ruler representing age, the patient will receive 61 points. The absence of ascites results in zero points. Due to the blood flow in the papillary structures, the patient receives 33 points. In a similar way 80 points are gained for the diameter of the solid component, 27 for the irregular cyst wall and 84 for the absence of shadows. In total, this patient receives 285 points. To obtain the risk of malignancy, a line needs to be drawn between the total points ruler at 285 points towards the risk of malignancy ruler. The patient specific risk of malignancy is nearly 90%.

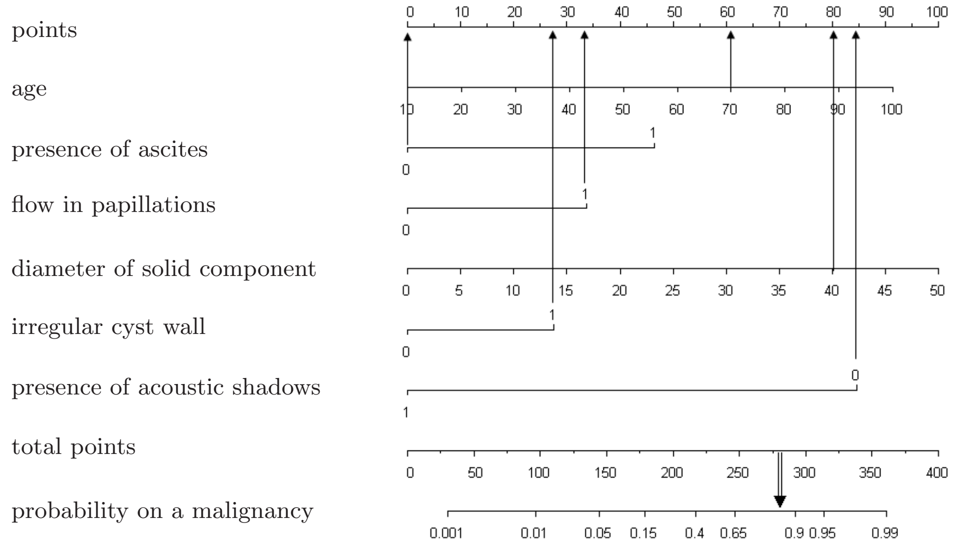

Fig. S1: Nomogram for the diagnosis of ovarian cancer derived from a logistic regression model (LR2 in [2]). For a paper-based implementation, the clinician has to draw vertical lines from the value of each variable to the point axis (upward arrows). The obtained points need to be added to get the total number of points for the patient. The risk of a malignant mass is found by drawing a vertical line from the total number of points to the risk of malignancy axis (see downward arrow from the total points axis to the risk axis).

Illustration of a model implementation created with the ICS methodology

This Section gives further explanation on the video in movie S1. This application was implemented in Microsoft Excel. It might be incorporated in software packages, touchscreen applications and included in clinical decision support systems. Starting from an empty screen, the user has to go to the *data entry* sheet and start the application by clicking the *Click here to start the application* button. An application sheet will pop up and the user can click on the intervals that apply to his/her patient. When more than one, or none of the intervals for a variable is marked, an error will appear on the screen. Only when all questions of Table 3 in the manuscript are answered, the user will receive a risk calculation after clicking the *calculate risk* button. A new window pops up indicating the total score and the corresponding risk. This window is closed by clicking the *close result* button. A click on the *clear form* button brings the user back to the initial Excel sheet. The results are stored in the *ovarian* sheet. For each variable, the allocated points are given and the total number of points is represented together with the associated risk. Other possible implementations are provided in movie S2 and movie S3.

Software

The proposed method was implemented as a convex optimization problem [3] in Matlab*, using CVX†.

* http://www.mathworks.com/products/matlab/

† http://cvxr.com/cvx/

References

1. Siddique J, Harel O (2009) MIDAS: A sas macro for multiple imputation using distance-aided selection of donors. Journal of Statistical Software 29: 1-18.
2. Timmerman D, Testa AC, Bourne T, Ferrazzi E, Ameye L, et al. (2005) Logistic regression model to distinguish between the benign and malignant adnexal mass before surgery: a multicenter study by the international ovarian tumor analysis group. Journal of Clinical Oncology 23: 8794-8801.
3. Boyd S, Vandenberghe L (2004) Convex optimization. Cambridge University Press, Cambridge.
